# Gene Dosage, Expression, and Ontology Analysis Identifies Driver Genes in the Carcinogenesis and Chemoradioresistance of Cervical Cancer

**DOI:** 10.1371/journal.pgen.1000719

**Published:** 2009-11-13

**Authors:** Malin Lando, Marit Holden, Linn C. Bergersen, Debbie H. Svendsrud, Trond Stokke, Kolbein Sundfør, Ingrid K. Glad, Gunnar B. Kristensen, Heidi Lyng

**Affiliations:** 1 Department of Radiation Biology, Norwegian Radium Hospital, Oslo, Norway; 2 Norwegian Computing Center, Oslo, Norway; 3 Department of Mathematics, University of Oslo, Oslo, Norway; 4 Department of Gynecologic Oncology, Norwegian Radium Hospital, Oslo, Norway; 5 Department of Medical Informatics, University of Oslo, Oslo, Norway; RIKEN Genomic Sciences Center, Japan

## Abstract

Integrative analysis of gene dosage, expression, and ontology (GO) data was performed to discover driver genes in the carcinogenesis and chemoradioresistance of cervical cancers. Gene dosage and expression profiles of 102 locally advanced cervical cancers were generated by microarray techniques. Fifty-two of these patients were also analyzed with the Illumina expression method to confirm the gene expression results. An independent cohort of 41 patients was used for validation of gene expressions associated with clinical outcome. Statistical analysis identified 29 recurrent gains and losses and 3 losses (on 3p, 13q, 21q) associated with poor outcome after chemoradiotherapy. The intratumor heterogeneity, assessed from the gene dosage profiles, was low for these alterations, showing that they had emerged prior to many other alterations and probably were early events in carcinogenesis. Integration of the alterations with gene expression and GO data identified genes that were regulated by the alterations and revealed five biological processes that were significantly overrepresented among the affected genes: apoptosis, metabolism, macromolecule localization, translation, and transcription. Four genes on 3p (*RYBP*, *GBE1*) and 13q (*FAM48A*, *MED4*) correlated with outcome at both the gene dosage and expression level and were satisfactorily validated in the independent cohort. These integrated analyses yielded 57 candidate drivers of 24 genetic events, including novel loci responsible for chemoradioresistance. Further mapping of the connections among genetic events, drivers, and biological processes suggested that each individual event stimulates specific processes in carcinogenesis through the coordinated control of multiple genes. The present results may provide novel therapeutic opportunities of both early and advanced stage cervical cancers.

## Author Summary

Genetic gains and losses, i.e. changes in gene dosages, are common abnormalities of human cancers. Discovering these defects and understanding the biological meaning can lead to improved therapeutic opportunities. This paper reports a large scale screening of gene dosage alterations in cervical cancer and gives a broader exploration of the expression and function of genes with gains or losses. We have focused on the most frequent gene dosage alterations and the alterations associated with survival after chemoradiotherapy, since these defects are likely to be of major importance for developing disease. The most notable finding was the discovery of a set of biological processes that are known hallmarks of cancer and were associated with gains and losses of specific genes. Moreover, novel loci associated with chemoradioresistance independent of existing clinical markers were found, and the genes involved were depicted. Our results indicated that gene dosage alterations play a causative role in the carcinogenesis and chemoradioresistance of cervical cancer and pinpointed candidate biomarkers of the disease.

## Introduction

Cervical cancer is one of the most common malignancies affecting women worldwide and a major cause of cancer death for women globally [1]. Radiotherapy combined with cisplatin is the treatment of choice at the locally advanced stages [2]. Improved therapy is needed, since more than 30% of the patients show progressive disease within 5 years after diagnosis and treatment related side effects to organs within the pelvis are frequent. Tumor stage, size, and lymph node involvement are the most powerful markers of aggressive disease, but do not fully account for the observed variability in outcome and are not biologically founded. A better handling of the disease may be provided by the discovery of efficient biomarkers for therapeutic planning and intervention, but requires more insight into the mechanisms underlying cervical carcinogenesis and treatment relapse.

During carcinogenesis, genetic and epigenetic alterations drive the evolution of tumor towards increased malignancy and treatment resistance. The changes enable tumor cells to overcome microenvironmental constraints, sustain proliferation, and invade adjacent tissues and distinct organs [3]–[5]. Gene dosage alterations like gains and losses regulate the expression of genes and are motive forces for this evolution [6],[7]. Tumor cells bearing an increasing number of gains and losses successively emerge and are selected for based on the growth advantage caused by the genetic changes. Discovery and functional assessment of gene dosage alterations involved in carcinogenesis are therefore essential for understanding the biology of the disease.

At the locally advanced stages of cervical cancer, numerous gene dosage alterations and severe aneuploidy are frequently seen [8]–[10]. Moreover, pronounced intratumor heterogeneity in the gains and losses exists within the tumors, reflecting a high genetic instability [9]. The consequences of these alterations for the tumor phenotype are difficult to predict, since large chromosomal regions involving multiple genes are generally affected and some aberrations may be random events without biological significance [11]. Genome wide screening of DNA copy numbers in a decent number of patients enables identification of recurrent gene dosage alterations; *i.e.*, alterations characteristic of the disease, and alterations associated with the clinical outcome [12], which are likely to be important in carcinogenesis and treatment resistance. Combining the data with expression profiles of the same tumors reveals the genes that are regulated primarily by the genetic events. The potential of this integrative strategy was recently demonstrated in a study on 15 early stage cervical cancers, where genes affected by aberrations on 1q, 3q, 11q, and 20q were reported [13]. Genetic events promoting tumor evolution and treatment resistance have, however, not been explored on a genome wide scale, and their biological meaning has not been addressed.

The present work was conducted to discover candidate driver genes and assess their function in the carcinogenesis and chemoradioresistance of cervical cancers. Genome wide screening of DNA copy numbers and expressions was performed in 102 patients with locally advanced disease. Of these, pairwise data were available for 95 patients. Reliable comparison of gains and losses across the patients was ensured by using the tumor ploidy, as determined by flow cytometry, and the GeneCount method to correct for the normal cell content of the samples and extract the absolute copy numbers and thereby the gene dosages [14]. The use of GeneCount also enabled mapping of the intratumor heterogeneity in the gene dosage alterations, providing information of the chronological order in which they had occurred during tumor evolution [14]. The recurrent gene dosage alterations, the alterations associated with outcome after chemoradiotherapy, and the genes that were regulated by these alterations were identified. Further analysis of gene ontology (GO) categories [15] was performed to identify biological processes that were overrepresented among the affected genes and therefore probably regulated by the gene dosage alterations. Such large scale and combined genomic, transcriptional, and functional analysis is powerful in detection of driver genes and their biological meaning, but has not been presented before. We demonstrate the potential of this approach by the identification of five biological processes in carcinogenesis that were associated with recurrent and predictive gains and losses of a set of genes. The set included four genes within the predictive losses for which repressed expression was related to poor outcome in the patient group and in an independent cohort of 41 patients. The genes are candidate drivers of the genetic events and novel biomarkers of cervical cancers.

## Results

### Recurrent Gene Dosage Alterations

**Figure 1:**
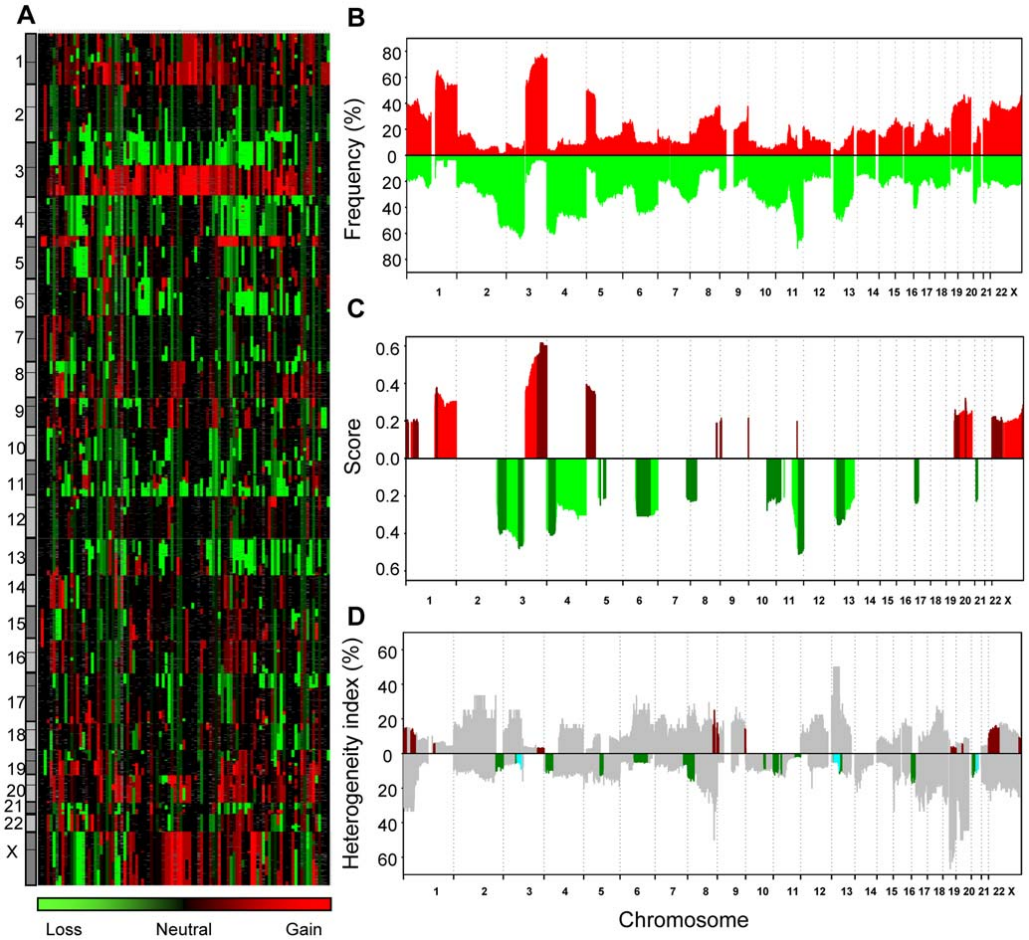
Gene dosage alterations of locally advanced cervical cancers. (A) Absolute gene dosage profile of 97 patients. Patients are shown in columns and gene dosages are ordered by DNA location in rows. The color scale ranges from green (loss) through black (neutral) to red (gain). Grey indicates missing values. (B) Frequency of patients with gains (red) and losses (green) along chromosome 1 to X for the patients in (A). Gene dosage alterations above 1.1 and below 0.9 were classified as gains and losses, respectively. (C) Score of recurrent gains (red) and losses (green) along chromosome 1 to X for the patients in (A). Peak regions, defined in Table 2, are shown in darker colors. (D) Intratumor heterogeneity in gene dosage alterations along chromosome 1 to X for the patients in (A). The heterogeneity index is shown for gains (above the zero line) and losses (below the zero line) separately, and was calculated as the number of heterogeneous cases relative to the total number of cases with alteration at each DNA location. The peak regions shown in (C) are marked in red (recurrent gains) and green (recurrent losses). The predictive losses are indicated in light blue.

Cervical cancer patients subjected to curative chemoradiotherapy were included in the study (Table 1). Most cases were squamous cell carcinoma and human papillomavirus (HPV) positive. Aneuploidy was seen in about half of the tumors, including some of the adenosquamous carcinomas and HPV negative cases (Figure S1A, S1B). Based on 97 patients, we generated an absolute gene dosage profile of the cancer genome by the use of array comparative genomic hybridization (aCGH) and the GeneCount analysis tool (Figure 1A). All chromosomes were affected with gains and losses, however, some regions were more frequently found to be aberrant than others (Figure 1B). Clustering of the patients based on gene dosages revealed no clear groups with characteristic aberrations.

**Table 1:** Patient and tumor characteristics.

Characteristic	Basic cohort (n = 102)	Validation cohort (n = 41)
Histology (n)		
Squamous	96	40
Adenocarcinoma	1	0
Adenosquamous carcinoma	5	1
HPV status (n)^a^^,^ b		
HPV16	65	35
HPV18	7	0
HPV16+18	11	1
HPV other	10	4
HPV negative	8	1
FIGO stage (n)		
1B	6	2
2	57	27
3	35	9
4A	4	3
Tumor size^c^: vol (cm^3^)^d^, diameter (cm)^e^		
Median	45.1, 4.4	36.6, 4.1
Range	2.8–321, 1.8–8.5	8.7–192, 2.5–7.2
Pelvic lymph node status^c^ (n)		
Positive	37	12
Negative	65	29
Age (years)		
Median	56	55
Range	28–85	25–81
Observation time (months)		
Median	42	31
Range	21–71	24–46
Relapse	32	12

a PCR on DNA was performed, using the primers listed in [9]. The products were detected by polyacrylamide gel electrophoresis or the Agilent DNA 1000 kit (Agilent Technologies Inc., Germany).

b HPV status was not determined for one patient in the basic cohort due to lack of DNA for analysis.

c Tumor size and lymph node status were determined from pretreatment magnetic resonance (MR) images.

d Volume was calculated based on 3 orthogonal diameters (a,b,c) as (π/6)*abc.

e Diameter was calculated from tumor volume (4π/3)*r^3^.

The recurrent gains and losses were identified by considering both the amplitude and frequency of each alteration in Figure 1B [16]. Hence, a larger weight was given to high-amplitude events that are less likely to be random aberrations without biological significance. The recurrent alterations comprised more than 42% of the genome, and consisted of 14 regions (528 Mb) with gain and 15 (734 Mb) with loss (Figure 1C). Most of these alterations were also seen in the adenosquamous carcinomas and the HPV negative tumors (Figure S1C, S1D). The most common alterations were gain on 1q, 3q, 5p, 20q, and Xq and loss on 2q, 3p, 4p, 11q, and 13q, each involving 44–76% of the patients (Figure 1C, Table 2). High level amplification (seven regions) and homozygote deletion (six regions) helped to depict the peak of five recurrent gains and two recurrent losses (Table 2, Table S1). The frequency of the homozygote deletions was low (1–3%, Table S1), and none of the tumors had more than one of them. Homozygote alteration is therefore probably not a common mechanism of gene regulation in cervical cancers, in contrast to the highly frequent heterozygote deletion. The highest gene dosage of 36 was found in a diploid tumor with a copy number of 72 on 11q22.1-2 (Table 2).

**Table 2:** Gene dosage alterations and correlating genes in locally advanced cervical cancer.

Peak region^a^	Peak region^a^	Freq.^b^	Max./min. gene dosage^c^	Correlating genes^d^
(Cytoband)	(MB)	(%)	(copy no.)	
**Recurrent gain**				
1p36.21-pter	0–14.6	38	2 (4)	*SLC35E2*, *UBE4B*, *AGTRAP*
1p32.1-p34.3	37.3–59.9	40	2 (4)	*C1orf149*, *YRDC*, *RLF*, *EBNA1BP2*, *TACSTD2*
1q21.1-q22	148.0–153.7	61	2.5 (6)	*SF3B4*, *ENSA*, *GOLPH3L*, *ARNT*, *LASS2*, *ANXA9*, *POGZ*, *CGN*, *SNX27*, *C1orf77*, *ILF2*, *DENN4B*, *SLC39A1*, *UBE2Q1*, *EFNA1*, *KRTCAP2*, *MUC1*, *FDPS*
3q26.1-qter^e^	166.2–199.5	75	4.5 (9)	*PDCD10*, *PHC3*, *ZNF639*, *FXR1*, *PARL*, *DVL3*, *ABCF3*, *ALG3*, *EIF4G1*, *SFRS10*, *DGKG*, *EIF4A2*, *RFC4*, *CCDC50*, *PPP1R2*, *PAK2*, *NCBP2*, *DLG1*, *BDH1*, *FLYTTD1*
5p15.2-pter^e^	1.0–12.1	47	4 (15)	*CLPTM1L* , *MED10*, *FASTKD3*, *CCT5*, *DAP*
8q24.13-22	125.7–134.1	37	2 (4)	None
8q24.3-qter	144.5–146.3	38	2 (4)	*TSTA3*, *FAM83H*, *CYC1*
9p24.1-2^e^	2.7–6.0	22	13.5 (27)	*KIAA0020*, *RCL1*
9q34.2-qter	135.6–138.2	35	3.5 (7)	*MRPS2*
11q22.1-2^e^	100.2–102.0	14	36 (72)	*YAP1*, *BIRC3*, *BIRC2*
19q13.11-qter	40.3–63.8	36	10 (29)	*SPINT2*, *PSMD8*, *CAPN12*, *MRPS12*, *RPS16*, *AP2S1*, *KDELR1*, *NUP62*, *ATF5*, *NKG7*, *ZNF787*
20q11.21-22^e^	30.0–33.0	45	3.4 (9)	*POFUT1*, *KIF3B*, *MAPRE1*, *SNTA1*, *EIF2S2*, *AHCY*
Xp11.22-pter^f^	0–54.1	38	2.5 (5)	*SLC25A6*, *CD99*, *ARSD*, *PNPLA4*, *PRPS2*, *PIR*, *CXorf15*, *PHKA2*, *PDHA1*, *RPS6KA3*, *PRDX4*, *EIF2S3*, *USP9X*, *DDX3X*, *NDUFB11*, *UBA1*, *EBP*, *PLP2*, *JARID1C*, *SMC1A*, *HUWE1*
Xq28-qter	148.5–154.9	47	4 (8)	*NSDHL*, *BCAP31*, *IDH3G*, *IRAK1*, *TAZ*, *LAGE3*, *UBL4A*, *FAM34*, *MTCP1*
**Recurrent loss**				
2q33.3-qter	206.2–243.0	54	0.26 (1)	*NDUFS1*, *SPAG16*, *MREG*, *SMARCAL1*, *AAMP*, *WNT10A*, *ZFAND2B*, *ANKZF1*, *STK11IP*, *FARSB*, *ACSL3*, *HRB*, *SP100*, *EIF4E2*, *COPS8*, *HDAC4*, *MTERFD2*, *PPP1R7*
3p12.3-p14.2	60.9–81.6	61	0.26 (1)	*RYBP*, *GBE1*
4p13-p16.1	8.3–42.3	58	0.42 (1)	*WDR1*, *UBE2K*, *PDS5A*
5q13.2^g^	67.4–71.7	38	0 (0)	*SMN2*
5q14.2-q15	82.5–96.9	35	0.5 (1)	*COX7C*, *TTC37*, *GLRX*
6q12-q23.2	67.0–132.9	42	0.43 (1)	*LMBRD1*, *MYO6*, *HMGN3*, *SYNCRIP*, *MAP3K7*, *CCNC*, *C6orf203*, *FOXO3*, *AMD1*, *HDAC2*, *NT5DC1*, *DSE*, *NUS1*, *ECHDC1*
7q34-qter	139.3–158.8	35	0.43 (1)	*PDIA4*
8p12-pter	0–31.9	32	0.34 (1)	*XPO7*, *BIN3*, *BNIP3L*, *EPHX2*, *CCDC25*, *DCTN6*, *PPP2CB*
10q23.31^g^	88.2–92.1	38	0 (0)	None
11p14.3-pter	0–24.4	40	0.5 (1)	*COPB1*, *PSMA1*, *GTF2H1*, *TSG101*
11p12	37.8–40.2	37	0.5 (1)	None
11q22.3-qter	105.1–134.5	63	0.35 (1)	*PPP2R1B*, *C11orf57*, *TIMM8B*, *REXO2*, *C11orf60*, *TRAPPC4*, *H2AFX*, *POU2F3*, *ARHGEF12*, *SC5DL*, *ZNF202*, *CHEK1*, *APLP2*, *ZBTB44*, *SNX19*
13q12.2-q21.32	27.5–67.4	46	0.33 (1)	*ALG5*, *FAM48A*, *COG6*, *KIAA1704*, *GTF2F2*, *MED4*, *RNASEH2B*
17p11.2-pter	0–19.1	38	0.27 (1)	*SPAG7*, *MPDU1*, *LSMD1*, *CYB5D1*, *COPS23*
21q21.1-3	18.3–28.6	35	0.28 (1)	*ATP5J*
**Predictive loss**				
3p11.2-p14.1	67.0–87.6	58	0.26 (1)	*RYBP*, *GBE1*
13q13.1-q21.1	30.0–56.5	46	0.41 (1)	*ALG5*, *FAM48A*, *COG6*, *KIAA1704*, *GTF2F2*, *MED4*, *RNASEH2B*
21q22.2-3	38.0–46.4	23	0.28 (1)	*PCP4*, *RIPK4*, *PDXK*

a Peak region of the recurrent gains and losses is the minimum shared region surrounded by at least three patients. In cases of recurrent high level amplification or homozygote deletion, this event determines the peak region. Peak region of the predictive losses is the region selected by LASSO.

b Frequency is the median percentage of tumors with the alteration.

c Gene dosage is absolute DNA copy number divided by ploidy. Maximum (gain) or minimum (loss) gene dosage and corresponding copy number are listed.

d Genes within the peak region showing a correlation between gene dosage and expression are ordered by DNA location.

e Recurrent high level amplification detected within recurrent gain. Peak region is the region with more than 25% higher amplitude than surrounding region.

f Probably two different peak regions.

g Homozygote deletion within recurrent loss. Peak region is the region with a gene dosage of zero.

#### Intratumor heterogeneity of the recurrent alterations

Intratumor heterogeneity in one or more of the gene dosage alterations was seen in about half of the patients [14]. The ploidy and genetic alterations of the heterogeneous tumors were similar to that of the homogeneous ones (Figure S2). It is reasonable to assume that homogeneous alterations have emerged before the heterogeneous ones during tumor evolution [9]. To order the recurrent alterations chronologically in relation to the less common alterations, we therefore mapped the intratumor heterogeneity along the chromosomes based on the absolute data achieved with GeneCount [14]. The heterogeneity was low for the recurrent alterations compared to others, like gain on 2q and 13q and loss on 1q, 19q, and 20q (Figure 1D). The recurrent aberrations had therefore probably occurred prior to many of these less common events.

### Gene Dosage Alterations in Relation to Outcome after Chemoradiotherapy

**Figure 2:**
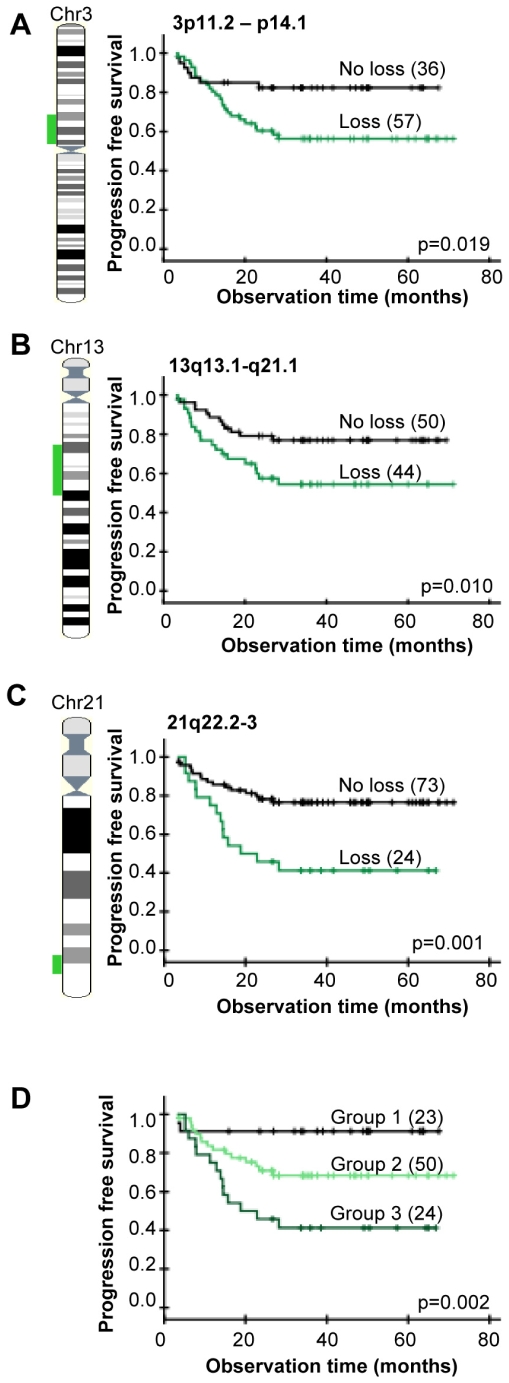
Gene dosage alterations and outcome after chemoradiotherapy. Kaplan-Meier curves of progression free survival for cervical cancer patients with (green) and without (black) loss of 3p11.2-p14.1 (A), 13q13.1-q21.1 (B), 21q22.2-3 (C), and for patients with different combinations of the three losses (D). P-values in log-rank test and number of patients are indicated. Data of the most significant genomic clone within each region were used; *i.e*, BAC clone ID RP11-118O11 (3p), RP11-408L13 (13q), and RP1-128M19 (21q). Total number of patients in (A, B) is less than 97 due to missing gene dosage data. (A–C) The lost DNA region is indicated on the chromosome (*left*). (D) Group 1: patients without loss of 3p11.2-p14.1, 13q13.1-q21.1, or 21q22.2-3, group 2: patients with loss of 3p11.2-p14.1 and/or 13q13.1-q21.1, but not 21q22.2-3, group 3: patients with loss of 21q22.2-3 only or loss of 21q22.2-3 combined with loss of 3p11.2-p14.1 and/or 13q13.1-q21.1. The groups were determined from data of each possible combination of the losses (Figure S3).

Gene dosage alterations responsible for poor clinical outcome may not be as common as the recurrent ones. All alterations in Figure 1B were therefore included in the survival analysis. The LASSO method identified three regions with loss, 3p11.2-p14.1, 13q13.1-q21.1, and 21q22.2-3, which jointly showed the strongest association to progression free survival (Table 2). The 3p11.2-p14.1 and 13q13.1-q21.1 regions overlapped with the recurrent 3p12.3-p14.2 and 13q12.2-q21.32 losses, whereas the predictive loss of 21q22.2-3 was distal of the recurrent loss of 21q21.1-3. The predictive losses were not correlated and were related to poor outcome also when analyzed separately (Figure 2A–2C). The intratumor heterogeneity of the losses was low and similar to that of the recurrent losses (Figure 1D).

Most patients had more than one of the predictive 3p, 13q, and 21q losses. We therefore investigated whether there was an increased risk of relapse in cases of two or three losses. Kaplan-Meier plots for patients with different combinations of the predictive losses revealed three major groups with different outcome (Figure S3). Patients without any of the losses had a low risk of relapse and a survival probability of 91% (Figure 2D). Patients with 3p and/or 13q loss, without 21q loss, had an intermediate survival probability of 68%, whereas those with 21q loss had the lowest survival probability of 44%. The risk of relapse therefore seemed to be particularly high when loss of 21q22.2-3 was involved.

**Figure 3:**
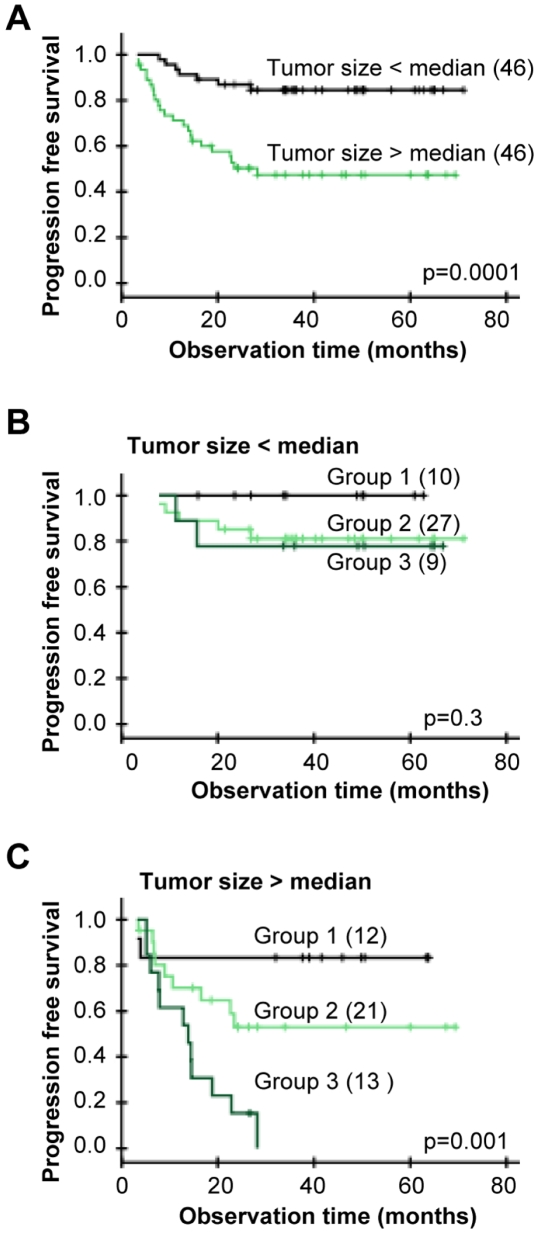
Gene dosage alterations and outcome after chemoradiotherapy for patients with different tumor size. (A) Kaplan-Meier curves of progression free survival for cervical cancer patients with tumor size above (green) and below (black) median. Ninety-two patients with tumor size determined from diagnostic MR images were included. Median size was 45.1 cm^3^, corresponding to a diameter of 4.4 cm. (B,C) Kaplan-Meier curves for patients in (A) with tumor size below median (B) and above median (C). Group 1: patients without loss of 3p11.2-p14.1, 13q13.1-q21.1, or 21q22.2-3, group 2: patients with loss of 3p11.2-p14.1 and/or 13q13.1-q21.1, but not 21q22.2-3, group 3: patients with loss of 21q22.2-3 only or loss of 21q22.2-3 combined with loss of 3p11.2-p14.1 and/or 13q13.1-q21.1. The groups were determined from data of each possible combination of the losses (Figure S3). P-values in log-rank test and number of patients are indicated.

The predictive impact of the 3p, 13q, and 21q losses were assessed by multivariate analysis together with tumor size, stage, and lymph node status. Histological type, HPV status, and heterogeneity status showed no correlation to outcome in univariate analysis and were therefore not included. The losses and tumor size had independent predictive value (Table 3), showing that the gene data contained information of the progression free survival that was not covered by tumor size. Since tumor size is a strong predictor (Figure 3A), we also investigated the predictive impact of the three losses for small and large tumors separately. About 20% of the patients with tumor size less than the median had relapse and all of them had one or more of the losses (Figure 3B). In the cases of tumors larger than the median, about 47% of the patients progressed and all except two of them had one or more of the losses (Figure 3C). None of the patients with loss involving 21q were disease free after 28 months, suggesting a particularly high risk of relapse in cases of a large tumor bearing loss of 21q22.2-3. There was no difference in tumor size for patients with and without loss in Figure 3B or in Figure 3C (data not shown). The gene data therefore enabled identification of high and low risk patients both in cases of a small and a large tumor.

**Table 3:** Cox regression analysis of genetic losses and clinical variables.

	Univariate analysis^a^			Multivariate analysis^a^		
Covariate	P	HR	95% CI	P	HR	95% CI
Loss of 3p11.2-p14.1^b^	0.003	0.27	0.11–0.66	0.018	0.33	0.13–0.83
Loss of 13q13.1-q21.1^b^	0.006	0.32	0.14–0.72	0.015	0.35	0.14–0.82
Loss of 21q22.2-3^b^	0.004	0.34	0.16–0.71	0.019	0.32	0.12–0.84
Tumor size^c^	0.001	4.5	1.9–10.5	0.001	5.5	1.9–15.5
FIGO stage^d^	0.004	2.9	1.4–5.9	0.072	-	-
Total lymph node status^e^	0.030	0.46	0.22–0.93	0.285	-	-

a P-value (P), hazard ratio (HR), and 95% confidence interval (CI) are listed.

b Semi-discrete gene dosage data of the most significant genomic clone within each region were used.

c Tumor size was divided in two groups based on the median size of 45.1 cm^3^, corresponding to a median diameter of about 4.4 cm.

d FIGO stage was divided in two groups; 1b–2b and 3a–4a.

e Total includes pelvic and para aortal lymph nodes.

### Integration of Gene Expression

To find genes regulated by the recurrent and predictive gene dosage alterations, we used cDNA microarrays and generated a cancer gene expression profile. The profile was based on 100 patients, including 95 of those analyzed with aCGH. Expression data were available for 1357 of the about 4000 known genes within the altered regions, and a significant correlation to gene dosage was found for 191 of them (Table 2). Several correlating genes were identified for each region, except for 8q24.13-22, 10q23.31, and 11p12, where no genes were found. Typical examples of correlation plots are shown in Figure S4. The results were confirmed with the Illumina gene expression assay on 52 patients. Although the Illumina analysis was based on a lower number of patients, an excellent correlation between the Illumina and cDNA data and between the Illumina and gene dosage data was found for almost all of the genes, as demonstrated in Table S2. We also performed a second cDNA analysis, including only tumors with more than 70% tumor cells in hematoxylin and eosin (HE) stained sections. Totally 179 of the genes (94%) were identified, suggesting few false positive results due to normal cells in the samples. The observations supported our conclusion that the genes in Table 2 were gene dosage regulated. The latter analysis identified 26 genes that were not depicted when all patients were considered. These genes were not considered further, since the results were based on only half of the data set.

Expression of known oncogenes and tumor suppressor genes within the depicted regions, like *MYC* (8q24.21), *BRCA2* (13q13.1), *RB1* (13q14.2), and *TP53* (17p13.1), was not significantly correlated to gene dosage. These genes are therefore probably not regulated primarily by gains and losses. The *TP53* and *RB1* results were consistent with the high frequency of HPV positive tumors (Table 1).

**Figure 4:**
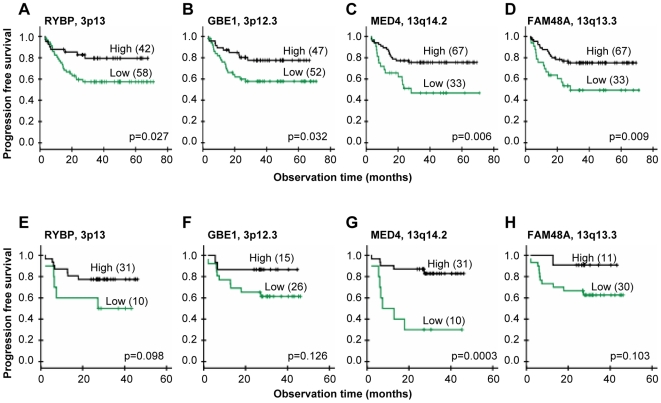
Gene expressions and outcome after chemoradiotherapy. Kaplan-Meier curves of progression free survival for cervical cancer patients with low (green) and high (black) expression of *RYBP* (A,E), *GBE1* (B,F), *MED4* (C,G), and *FAM48A* (D,H). cDNA data of 100 patients is used in (A–D), and Illumina data of an independent cohort of 41 patients is used in (E–H) for validation. P-value in log-rank test and number of patients are indicated. The number of patients in each group was chosen to achieve the largest difference in survival between the groups, approximately reflecting the number of patients with and without loss in (A–D). Total number of patients is less than 100 in (B) due to missing gene expression data.

The predictive losses on 3p and 13q involved the same correlating genes as the corresponding recurrent ones, whereas *PCP4*, *RIPK4*, and *PDXK* were correlating genes within the predictive 21q region (Table 2). To depict the correlating genes that most probably were involved in development of chemoradioresistance, we required that the gene was significantly associated with clinical outcome both at the gene dosage and expression level. Moreover, a clear difference in the survival curves should also be seen in an independent cohort of 41 patients when based on the Illumina gene expression data. The criteria were fulfilled for four genes; *RYBP* and *GBE1* on 3p and *MED4* and *FAM48A* on 13q, which were termed predictive genes (Figure 4). Two more genes, *GTF2F2* and *RNASEH2B* on 13q, were correlated to outcome based on the cDNA data, but were not considered further since the tendency based on the Illumina data was weak (p>0.15). The relationship to outcome was not strong enough for *PCP4*, *RIPK4*, and *PDXK* on 21q to be included among the predictive genes either.

### Gene Ontology Analysis

Biological processes associated with the recurrent and predictive gene dosage alterations were found by comparing the GO categories of the affected genes with those of all genes in the data set [15]. One or more biological processes were annotated to 155 of the correlating and predictive genes and to 5824 of all genes. The categories apoptosis, carbohydrate metabolism, translation, and RNA-protein complex biogenesis and assembly were significantly overrepresented among the correlating genes within the recurrent gains, whereas macromolecule localization, generation of precursor metabolites and energy, transcription from RNA polymerase II promoter, and establishment or maintenance of chromatin architecture were overrepresented among those within the recurrent and predictive losses (Table 4). Fifty-six genes were included in the significant categories and were candidate drivers of the biological processes. In addition, we included the predictive gene *FAM48A*, which was not associated to any process in the GO database, as a potential driver of chemoradioresistance together with *RYBP* and *MED4* (transcription) and *GBE1* (generation of precursor metabolites and energy).

**Table 4:** Biological processes overrepresented among the correlating genes within recurrent and predictive regions.

GO number	GO category	No. correlating genes	No. genes on the array	p-value	Correlating genes
Gains					
GO: 000815	Biological process	93^a^	5824^a^		
GO: 0006915	Apoptosis	13 (14.0%)	434 (7.5%)	0.026	*UBE4B*, *BIRC2*, *BIRC3* , *ATF5*, *BCAP31*, *CLPTM1L*, *DAP*, *FASTKD3*, *FXR1*, *NUP62*, *PAK2*, *PDCD10*, *SLC25A6*
GO: 0005975	Carbohydrate metabolism	7 (7.5%)	198 (3.4%)	0.038	*PPP1R2*, *ARNT*, *PHKA2*, *POFUT1*, *PDHA1*, *TSTA3*, *IDH3G*
GO: 0006412	Translation	7 (7.5%)	163 (2.8%)	0.015	*EIF4G1*, *EIF4A2*, *EIF2S2*, *MRPS12*, *RPS16*, *EIF2S3*, *MRPS2*
GO: 0022613	RNA-protein complex biogenesis and assembly	7 (7.5%)	89 (1.5%)	0.001	*EIF4G1*, *EIF4A2*, *EIF2S2*, *EIF2S3*, *EBNA1BP2*, *NCBP2*, *RCL1*
Losses					
GO: 000815	Biological process	62^a^	5824^a^		
GO: 0033036	Macromolecule localization	10 (16.1%)	427 (7.3%)	0.022	*BIN3*, *COPB1*, *COG6*, *XPO7*, *HRB*, *MYO6*, *PDIA4*, *SNX19*, *TIMM8B*, *TSG101*
GO: 0006091	Generation of precursor metabolites and energy	4 (6.5%)	117 (2.0%)	0.035	*ATP5J*, *COX7C*, *GBE1*, *NDUFS1*
GO: 0006366	Transcription from RNA polymerase II promoter	10 (16.1%)	357 (6.1)	0.004	*RYBP*, *FOXO3*, *GTF2F2*, *GTF2H1*, *MED4*, *MYO6*, *POU2F3*, *SMARCAL1*, *ZNF202*, *HDAC4*
GO: 0006325	Establishment or maintenance of chromatin architecture	5 (8.1%)	140 (2.4%)	0.016	*DSE*, *H2AFX*, *HDAC2*, *SMARCAL1*, *HDAC4*

a Genes with GO annotation (biological process).

**Figure 5:**
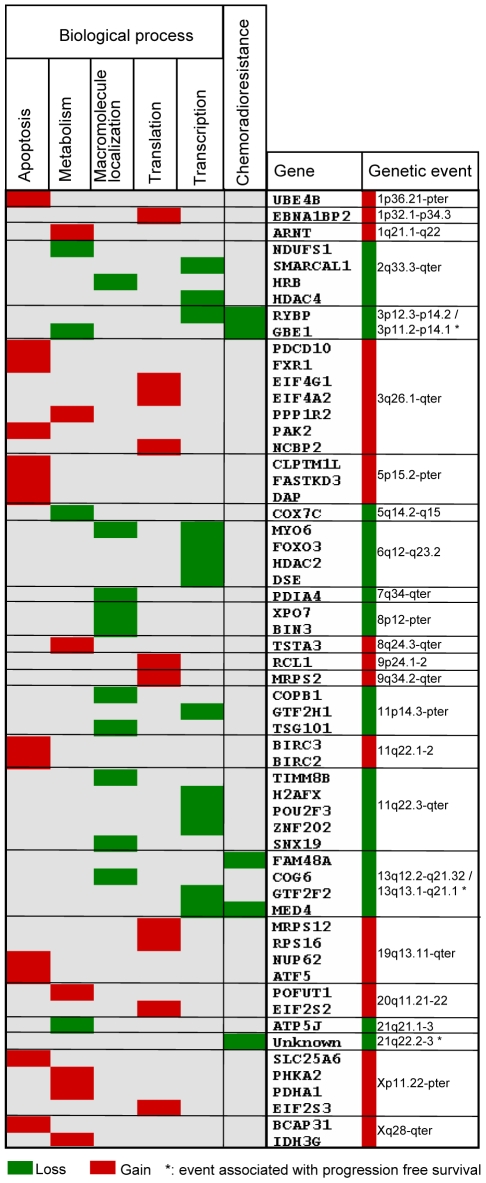
Genetic events, correlating genes, and biological processes in carcinogenesis and chemoradioresistance of cervical cancers. Recurrent and predictive gene dosage alterations, correlating genes, and biological processes overrepresented among the genes are listed. Only the genetic events associated with a process or chemoradioresistance (*) are included; six of the recurrent alterations are therefore not shown. The genes are ordered by DNA location. Correlating genes connected to chemoradioresistance were associated with clinical outcome both at the gene dosage and expression level and validated in an independent patient cohort. Gains and losses are indicated with red and green color, respectively.

We generated a map to visualize the connections between genetic events, affected genes, and biological processes (Figure 5). The processes carbohydrate metabolism and generation of precursor metabolites and energy were combined in metabolism, translation and RNA-protein complex biogenesis and assembly were combined in translation, and transcription from RNA polymerase II promoter was combined with establishment or maintenance of chromatin architecture in transcription. The combined categories were closely related, justifying this strategy. All but six of the recurrent alterations were associated with a process and represented in the map. The predictive 3p and 13q losses were merged with the corresponding recurrent losses, since the regions overlapped, and linked to metabolism (*GBE1*) and transcription (*RYBP*, *MED4*) in addition to chemoradioresistance. The predictive 21q loss was not connected to any known gene, but associated with chemoradioresistance. The map revealed features that seemed to be characteristic of recurrent and predictive alterations in cervical cancer. First, many of the genetic events were associated with clusters of genes in the same biological process. For example, gain on 3q affected three genes in apoptosis and three in translation, gain on 5p was linked to tree apoptosis genes, and loss on 6q was associated with four genes in transcription. Second, several events, like gain on 3q, 19q, 20q and loss on 2q, 6, and 11q, were connected to more than one biological process, either through the regulation of several genes or because some genes had multiple functions.

## Discussion

This work presents the first coupling of gene dosage and expression profiles in a large sample set of cervical cancers. We based our study on absolute gene dosages, which are more sensitive than the commonly used aCGH ratios in detecting gains and losses and enable comparisons across tumors with differences in ploidy and normal cell content [14]. This strategy and the large number of patients ensured reliable identification of recurrent gene dosage alterations, events associated with clinical outcome, and their intratumor heterogeneity. Further analysis based on GO categories provided an objective way of organizing the numerous correlating genes into biological meaningful information. We demonstrate a large potential of the integrative approach by the discovery and functional assessment of candidate driver genes that represent novel biomarkers of the disease. In particular, novel loci associated with clinical outcome were identified, providing the first evidence that gene dosage can be responsible for developing chemoradioresistance in cervical cancers.

The recurrent gene dosage alterations were consistent with earlier reports on advanced stage cervical cancer based on conventional CGH [8],[9],[17]. However, a more precise definition of the altered regions was achieved here due to the improved resolution of the array technique. The high frequency of the alterations suggests that they play a causative role in carcinogenesis. Hence, many of the alterations are common also in other squamous cell carcinomas, like head and neck cancers [18],[19]. Moreover, the recurrent loss on 3p and 13q overlapped with the losses associated with poor clinical outcome, strengthening the hypothesis of a central role in tumor evolution. Less frequent alterations can, however, also be crucial for tumor evolution, as was demonstrated by the recurrent gain on 11q22 in 14 patients and predictive loss on 21q in 23 patients.

The low intratumor heterogeneity of the recurrent and predictive gene dosage alterations indicated that they had occurred prior to many of the other alterations. The result was consistent with our previous cervical cancer study based on conventional CGH [9], showing a homogeneous intratumor distribution of the frequent gains on 3q, 5p, and 20q and losses on 3p and 11q14-qter. Moreover, regions overlapping with the 1p, 1q, 3q, 8q, 9q, and 20q recurrent gains and 2q, 3p, 4p, 11q, and 17p losses have been found to be altered in precancerous cervical intraepithelial lesions [17], [20]–[23], suggesting that the events had occurred at an early stage. It is therefore likely that the alterations identified here, and the consequently control of biological processes and development of chemoradioresistance, emerge early during carcinogenesis. It should be noted that a low heterogeneity was seen for some of the less common alterations as well, implying that they had occurred early. The affected genes in these regions may also be crucial for tumor evolution, however, other mechanisms than gene dosage alterations, such as epigenetic events or mutations, probably play the major role in their regulation. Moreover, some of the highly heterogeneous alterations may be important for disease progression a later stage, being a result of the continuing tumor evolution towards increased aggressiveness.

The gene dosage alterations were associated with specific biological processes that are closely related to known cancer hallmarks [3]–[5], indicating that the genes involved are drivers of carcinogenesis. Hence, gain of the genes in apoptosis, including the anti-apoptosis genes *BIRC2*, *BIRC3*, and *ATF5*, can help carcinoma cells to evade apoptosis [3]. Aberrations of the genes in metabolism, like gain of *ARNT* and *IDH3G* in carbohydrate metabolism, and loss of *COX7C* and *ATP5J* in oxidative phosphorylation, can be part of a metabolic reprogramming towards increased glycolysis and decreased mitochondrial function to meet the high energy demand linked to tumor growth [4]. In particular, gain of *ARNT* may increase hypoxia and hypoglycemia tolerance by signaling through the *HIF1A* pathway [24]. Loss of the genes in molecular localization, including *HRB* and *TSG101*, can lead to abnormal protein internalization and recycling and thereby abrogated degradation of proteins like growth factor receptors [25],[26]. Finally, aberrations of the genes in translation and transcription, such as gain of the translation initiation factors *EIF4A2*, *EIF4G1*, *EIF2S2*, and *EIF2S3* and loss of the transcriptional repressors *HDAC2* and *HDAC4*, can be a way to control the formation and activity of essential proteins. The EIF-proteins are central in adaptation to hypoxia and can stimulate *MYC* translation and thereby oncogenic processes like cell proliferation [27],[28]. Improper function of *HDAC2* and *HDAC4* may also increase proliferation [29]. Many of the genes, including *BIRC2*, *BIRC3*, *ATF5*, *NUP62*, *FASTKD3*, *IDH3G*, and *POFUTI*, have been found to be regulated by gains or losses in previous cervical cancer studies [30]–[33]. Our findings link each gene to one or more specific biological processes, and thereby indicate the functional meaning of the genetic events in carcinogenesis.

Loss and down regulation of *GBE1* and *RYBP* on 3p and *MED4* and *FAM48A* on 13q were associated with poor clinical outcome, suggesting that the genes are drivers of chemoradioresistance. The mechanisms underlying these findings and possible associations to known aggressive phenotypes like hypoxia and rapid proliferation [34]–[36] are not clear, but a tumor suppressor function of the genes has been indicated. *GBE1*, which plays a role in carbohydrate metabolism, has been found to be down regulated in ovarian cancers [37]. Loss of the transcriptional repressor *RYBP* may impair death receptor-mediated apoptosis [38],[39], and the encoded protein has been shown to be down regulated in many tumor types, including cervical cancer [40]. Loss of the transcriptional activators *MED4* may impair transcription of genes with anti-cancer effect, like the vitamin D receptor [41],[42]. The function of *FAM48A* is less clear, but some studies indicate that loss of this gene can promote aggressiveness. Hence, *FAM48A* is required for activation of the MAPK p38 pathway [43], which represses cell proliferation [44]. We found no candidate driver gene of chemoradioresistance within the predictive loss on 21q. Only a few tumor suppressor genes have been identified in this region. One candidate is the transcriptional regulator *PRDM15*, which was not included in our cDNA data set [45]. Our data showed, however, no correlation between *PRDM15* expression, assessed with the Illumina method in 52 patients, and gene dosage (data not shown), suggesting that the gene is not regulated by genetic loss. Further investigation with denser microarrays or possibly microRNA screening would be needed to find the drivers in this region.

The connection between genetic events, genes, and biological processes may provide insight into more general aspects of cervical carcinogenesis. Several genes were often associated with a single genetic event, supporting the hypothesis that there can be multiple drivers of an event that coordinately promote tumor evolution [11]. In cases of genes in the same biological process, like the anti-apoptosis genes *BIRC2* and *BIRC3* on 11q22, a broad and therefore efficient control of the process may be obtained. Hence, *BIRC2* and *BIRC3* may play complementary roles in apoptosis evasion, since upregulation of *BIRC3*, but probably not *BIRC2*, may impair hypoxia induced apoptosis [46],[47]. In cases of genes in different biological processes, such as metabolism (*NDUFS1*), macromolecule transport (*HRB*), and transcription (*SMARCAL1*, *HDAC4*) on 2q, the collective control of these processes through a single event is likely to give a growth advantage that is selected for in carcinogenesis. One or more genes in all biological processes were affected in most tumors due to the high frequency of the recurrent gene dosage alterations. All processes were therefore probably important, and the control of them through gains and losses seems to be a common feature of the disease.

The candidate driver genes represent novel biomarkers that may be utilized in the handling of cervical cancers. Diagnostic assessment of the biomarkers may help to understand the evolutionary status and therefore the biology of the cancer in individual patients. In particular, the predictive biomarkers may be used in addition to tumor size for classification of patients into risk groups in a personalized treatment regime. The biomarkers also open for the possibility to specifically repress biological processes in carcinogenesis by molecular targeting, and thereby interfere with tumor evolution. The use of drugs to inhibit translation by interaction with EIF-proteins has shown promising results [48] and been suggested as a tool to target tumor hypoxia [49]. The approach may be applied at all stages of the disease, since the genetic events probably emerge early. Moreover, improved outcome after chemoradiotherapy might be achieved by targeting the predictive biomarkers. Hence, viral-mediated delivery of *RYBP* has been shown to induce apoptosis in a number of cancer cell lines [38], and could be a useful strategy for the patients with loss of this gene.

## Materials and Methods

### Patients

A cohort of 102 patients was included for basic analyses to identify gene dosage alterations with aCGH (97 patients), affected transcripts with cDNA microarrays (100 patients), and to confirm the affected transcripts with the Illumina method (52 patients) (Table 1). An independent cohort of 41 patients was used to validate relationships between gene expression and outcome with the Illumina method (Table 1). All patients received external irradiation and brachytherapy combined with adjuvant cisplatin and were followed up as described previously [50]. Eighteen patients received extended radiation field due to enlarged common iliac and para-aortal lymph nodes. Progression free survival, defined as the time between diagnosis and the first event of locoregional and/or distant relapse, was used as end point. Six patients died of causes not related to cancer and were therefore censored. Tumor samples were collected at the time of diagnosis. One – four biopsies, approximately 5×5×5 mm in size, were taken at different locations of the tumor, immediately snap-frozen in liquid nitrogen and stored at −80°C until used for analyses. The study was approved by the regional committee of medical research ethics in southern Norway, and written informed-consent was achieved from all patients.

### Array Comparative Genomic Hybridization

The aCGH experiments and generation of absolute gene dosage profiles have been described previously for all 97 patients (ArrayExpress accession no. E-TABM-398) [14]. The array slides were produced at the Microarray Facility at the Norwegian Radium Hospital and contained 4549 unique genomic BAC and PAC clones that covered the whole genome with a resolution of approximately 1 Mb. Genomic DNA was isolated from the biopsies, labeled, and co-hybridized with normal female DNA to the array slides. DNA from different biopsies of the same tumor was pooled. The biopsies of all except two patients had more than 50% tumor cells in HE stained sections from the middle part of the sample. Median tumor cell fraction was 70% (range 30–90%). After array scanning, image analysis, spot filtering, and ratio normalization, the GLAD algorithm was applied for ratio smoothing and breakpoint detection [51].

#### Absolute gene dosages

The smoothed ratios were transferred to absolute DNA copy numbers in GeneCount by utilizing tumor ploidy data and correcting for the normal cell content of the samples [14]. The tumor ploidy was determined from a separate piece of the biopsy by flow cytometry, and tumor cell fraction was estimated by the program prior to the copy number calculations. The ploidy data and tumor cell fractions have been presented previously [14]. The tumor cell fractions, ranging from 27% to 84%, were in general lower than the results based on HE stained sections, probably because the amount of immune cells infiltrating the tumor parenchyma are difficult to quantify by histological examination [14]. The copy numbers were rounded off to the nearest integer values.

The absolute gene dosage profile of each tumor was generated by dividing each copy number by the ploidy. A gene dosage of 1 therefore implied no change in the copy number. The gene dosage thresholds for scoring gains and losses were 1.1 and 0.9, respectively, taking into account an uncertainty in the ploidy measurement of approximately 10%. For scoring high level amplification, a gene dosage of 2.5 or higher; *i.e.* 5 DNA copies in diploid tumors, was required. Homozygote deletions had a gene dosage of 0.

#### Intratumor heterogeneity

The intratumor heterogeneity in the copy numbers was assessed by comparing the aCGH ratio distributions of the possible heterogeneous regions with the distributions of the adjacent homogeneous regions by ANOVA analysis [14]. Totally 86 patients had a tumor cell fraction sufficiently high for reliable detection of heterogeneity, and the remaining eleven patients were excluded from this analysis. The heterogeneous regions have been listed previously [14]. A heterogeneity index was calculated for gains and losses separately, as the number of heterogeneous cases relative to the total number of cases with alteration at each DNA location. The copy number of the heterogeneous region was 0.5 above (gain) or below (loss) the nearest integer value.

The GeneCount method has been extensively validated based on the cervical cancer samples included in this study and a cohort of 94 lymphoma samples [14]. In particular, we used lymphoma samples to show that the estimated tumor cell fractions correlate significantly with the highly accurate values determined by flow cytometry [14].

### cDNA Microarrays

The cDNA microarray experiments have been presented previously for 48 of the 100 patients [50]. The array slides were produced at the Microarray Facility at the Norwegian Radium Hospital and contained more than 12000 unique cDNA clones, including most known oncogenes and tumor suppressor genes. Total RNA was isolated from the biopsies, labeled, and co-hybridized with reference RNA (Universal Human Reference RNA, Stratagene, La Jolla, CA) to the array slides. RNA from different biopsies of the same tumor was pooled. Only biopsies with more than 50% tumor cells in HE stained sections were utilized. Median tumor cell fraction was 70% (range 50–90%). All hybridizations were performed twice in a dye-swap design (ArrayExpress accession no. E-TABM-817). After array scanning, image analysis, spot filtering, and ratio normalization, the average expression ratios were calculated from the two data sets and used in the further analyses. The gene expressions were mapped to the gene dosages based on the exact chromosomal position of the cDNA and genomic clones, as derived from Ensembl (http://www.ensembl.org/Homo_sapiens/searchview).

### Illumina Gene Expression Beadarrays

Results based on cDNA data were validated with Illumina gene expression beadarrays in 52 of the patients subjected to aCGH and in the independent cohort of 41 patients. HumanWG-6 v3 beadchips (Illumina Inc., San Diego, CA) with 48000 transcripts were used. RNA was isolated from the biopsies as described above and amplified using the Illumina TotalPrep RNA amplification kit (Ambion Inc., Austin, TX) with 500 ng of total RNA as input material. cRNA was synthesized overnight (14 hr), labelled, and hybridized to the chips at 58°C overnight, according to the standard protocol. The hybridized chip was stained with streptavidin-Cy3 (AmershamTM, PA43001, Buckinghampshire, UK) and scanned with an Illumina beadarray reader. The scanned images were imported into BeadStudio 3.1.3.0 (Illumina Inc.) for extraction, quality control, and quintile normalization. The annotation file HumanWG-6_V3_0_R0_11282955_A was used.

### Statistics

The recurrent gene dosage alterations were identified based on a score that was calculated for each genomic clone by multiplying the average gene dosage amplitude with its frequency [16]. Gains and losses were considered in two separate procedures. Semi-discrete data were used, for which amplitudes lower than 1.1 were set to 1 when searching for gains and amplitudes higher than 0.9 were set to 1 when searching for losses. The score significance was assessed by comparison to similar scores obtained after data permutation [16], adjusting the p-value by a multiple testing procedure to control the false discovery rate (FDR) [52]. Recurrent alterations with an FDR q-value <5% were reported.

Gene dosage alterations associated with clinical outcome were identified with the LASSO method in the Cox proportional hazards model [53], as implemented in [54]. The LASSO is a method for variable selection and shrinkage in regression models when the number of covariates is larger than the number of individuals. By performing shrinkage in addition to selection, the LASSO is more stable than stepwise procedures where variables are either retained or discarded from the model sequentially, one at a time. In groups of highly correlated variables the LASSO tends to select only one variable in the group [55], and reported therefore one representative of each DNA region that jointly explained the outcome. Each region was thereafter found by selecting neighbouring genomic clones with strong correlation (r>0.9) to the one reported. Survival curves were generated by Kaplan-Meier analysis and compared by using log-rank test.

Spearman's rank correlation analysis with an FDR q-value <5% was used to search for significant correlations between gene dosage and expression. The analysis was based on semi-discrete data, retrieved as described above. To identify biological processes that were overrepresented among the correlating genes, the GO categories of the genes were compared with those of all genes on the array by using the master-target procedure with the Fisher's exact test in the eGOn software [15]. The GO categories were found in eGOn from public databases, based on the gene reporter EntrezGeneID.

## Supporting Information

Figure S1 Tumor ploidy and gene dosage alterations in relation to histological type and HPV status. (A) Ploidy distribution of 97 patients. Tumors with a ploidy within the range of 1.8–2.2 were considered as near diploid. (B) Ploidy of patients with adenosquamous carcinoma or HPV negative tumor. (C, D) Frequency of patients with gains (red) and losses (green) along chromosome 1 to X for patients with adenosquamous carcinoma (C) and HPV negative tumor (D). Gene dosage alterations above 1.1 and below 0.9 were classified as gains and losses, respectively. (A–D) Tumors in the basic cohort subjected to aCGH analysis were included.
(0.30 MB TIF)

Figure S2 Tumor ploidy and gene dosage alterations in homogeneous and heterogeneous tumors. (A) Ploidy distribution of patients with homogeneous (left) and heterogeneous (right) tumors. (B,C) Frequency of patients with gains (red) and losses (green) along chromosome 1 to X for patients with homogeneous (B) and heterogeneous (C) tumor. Gene dosage alterations above 1.1 and below 0.9 were classified as gains and losses, respectively. Totally 86 patients with a tumor cell fraction sufficiently high for reliable detection of heterogeneity were included in the analysis.
(0.29 MB TIF)

Figure S3 Clinical outcome for patients with different combinations of predictive losses. Kaplan-Meier curves showing progression free survival after chemoradiotherapy of 97 cervical cancer patients with different combinations of 3p11.2-p14.1, 13q13.1-q21.1, and 21q22.2-3 loss. The different combinations and number of patients in each group are listed (right). P-value in log-rank test is indicated.
(0.24 MB TIF)

Figure S4 Correlations between gene dosage and expression. Typical correlation plots of gene dosage and expression for 9 correlating genes within the recurrent and predictive regions; 6 with gain and 3 with loss. Spearman's rank correlation analysis on semi-discrete data was performed, for which amplitudes lower than 1.1 were set to 1 for gains and amplitudes higher than 0.9 were set to 1 for losses. Correlation coefficient (R) and p-value are indicated.
(0.27 MB TIF)

Table S1 Recurrent high-level amplifications and homozygous deletions in locally advanced cervical cancer.
(0.03 MB PDF)

Table S2 Relationships among Illumina, cDNA, and gene dosage data for correlating genes.
(0.07 MB PDF)
