# Supplementary figures and images for: Gene Dosage, Expression, and Ontology Analysis Identifies Driver Genes in the Carcinogenesis and Chemoradioresistance of Cervical Cancer
Source: PLoS Genet. 2009 Nov 13;5(11):e1000719. doi: 10.1371/journal.pgen.1000719 (PMC2768783; doi:10.1371/journal.pgen.1000719)

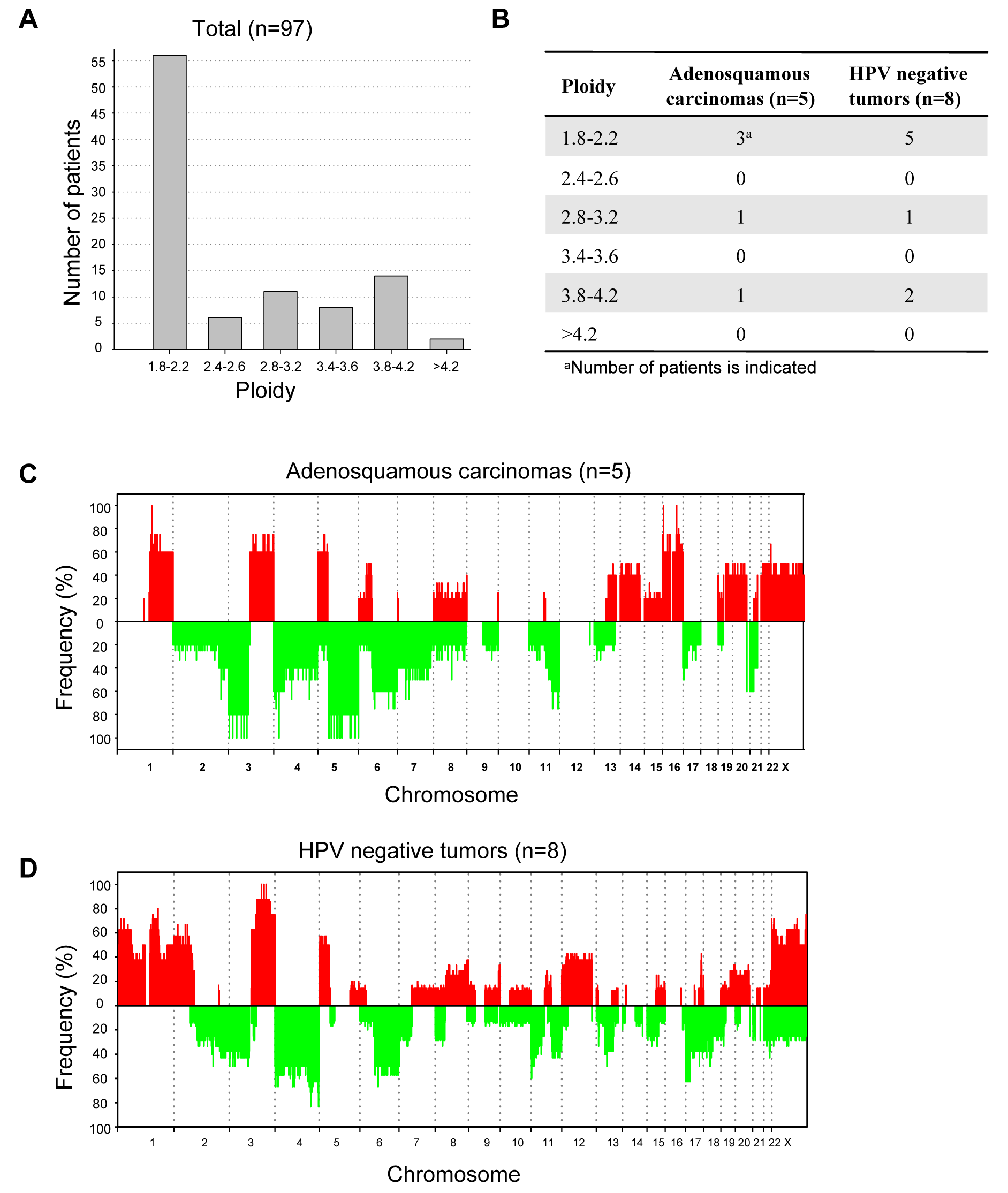

Supplement: Figure S1 — Tumor ploidy and gene dosage alterations in relation to histological type and HPV status. (A) Ploidy distribution of 97 patients. Tumors with a ploidy within the range of 1.8–2.2 were considered as near diploid. (B) Ploidy of patients with adenosquamous carcinoma or HPV negative tumor. (C, D) Frequency of patients with gains (red) and losses (green) along chromosome 1 to X for patients with adenosquamous carcinoma (C) and HPV negative tumor (D). Gene dosage alterations above 1.1 and below 0.9 were classified as gains and losses, respectively. (A–D) Tumors in the basic cohort subjected to aCGH analysis were included. (0.30 MB TIF) [file pgen.1000719.s001.tif]

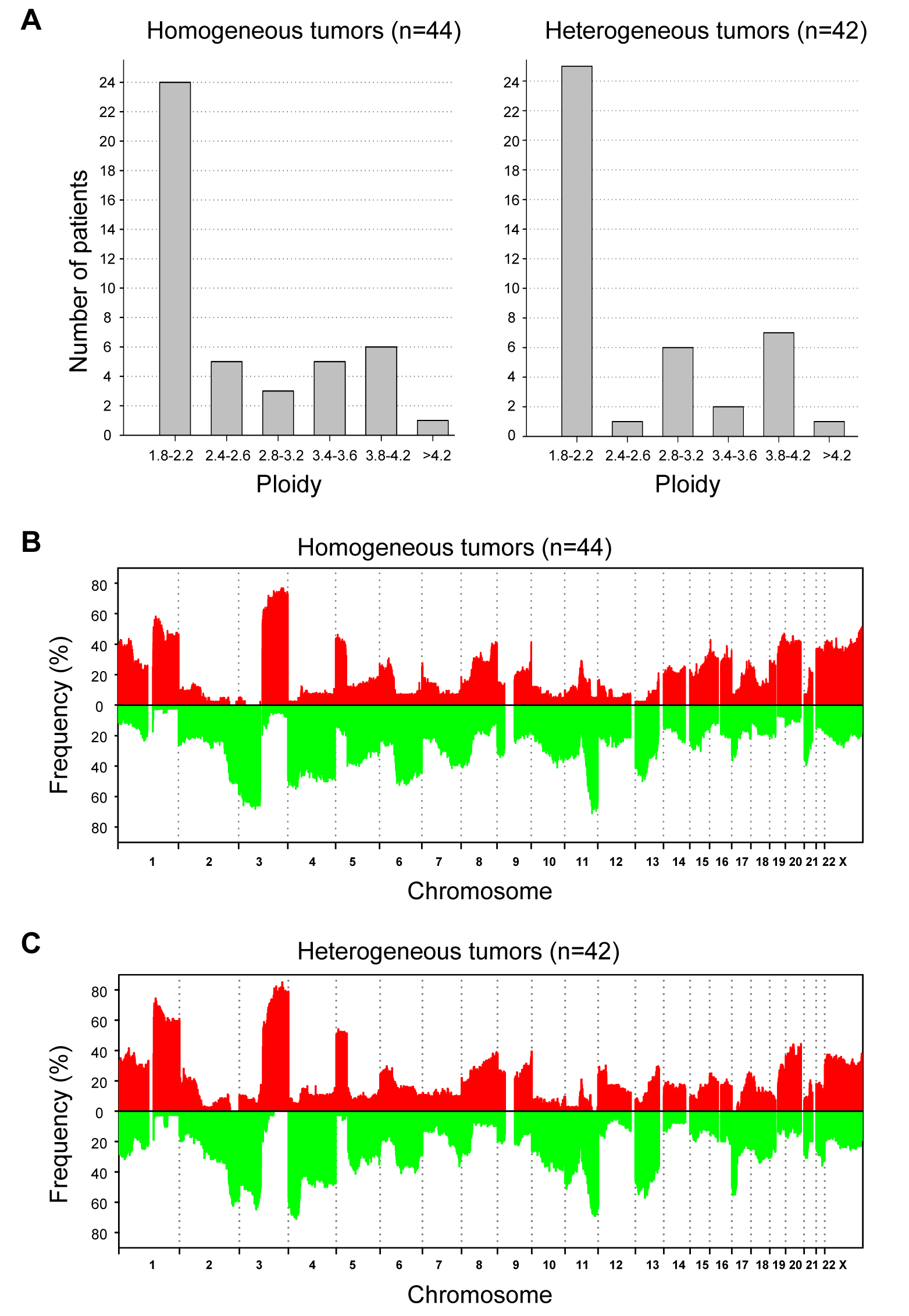

Supplement: Figure S2 — Tumor ploidy and gene dosage alterations in homogeneous and heterogeneous tumors. (A) Ploidy distribution of patients with homogeneous (left) and heterogeneous (right) tumors. (B,C) Frequency of patients with gains (red) and losses (green) along chromosome 1 to X for patients with homogeneous (B) and heterogeneous (C) tumor. Gene dosage alterations above 1.1 and below 0.9 were classified as gains and losses, respectively. Totally 86 patients with a tumor cell fraction sufficiently high for reliable detection of heterogeneity were included in the analysis. (0.29 MB TIF) [file pgen.1000719.s002.tif]

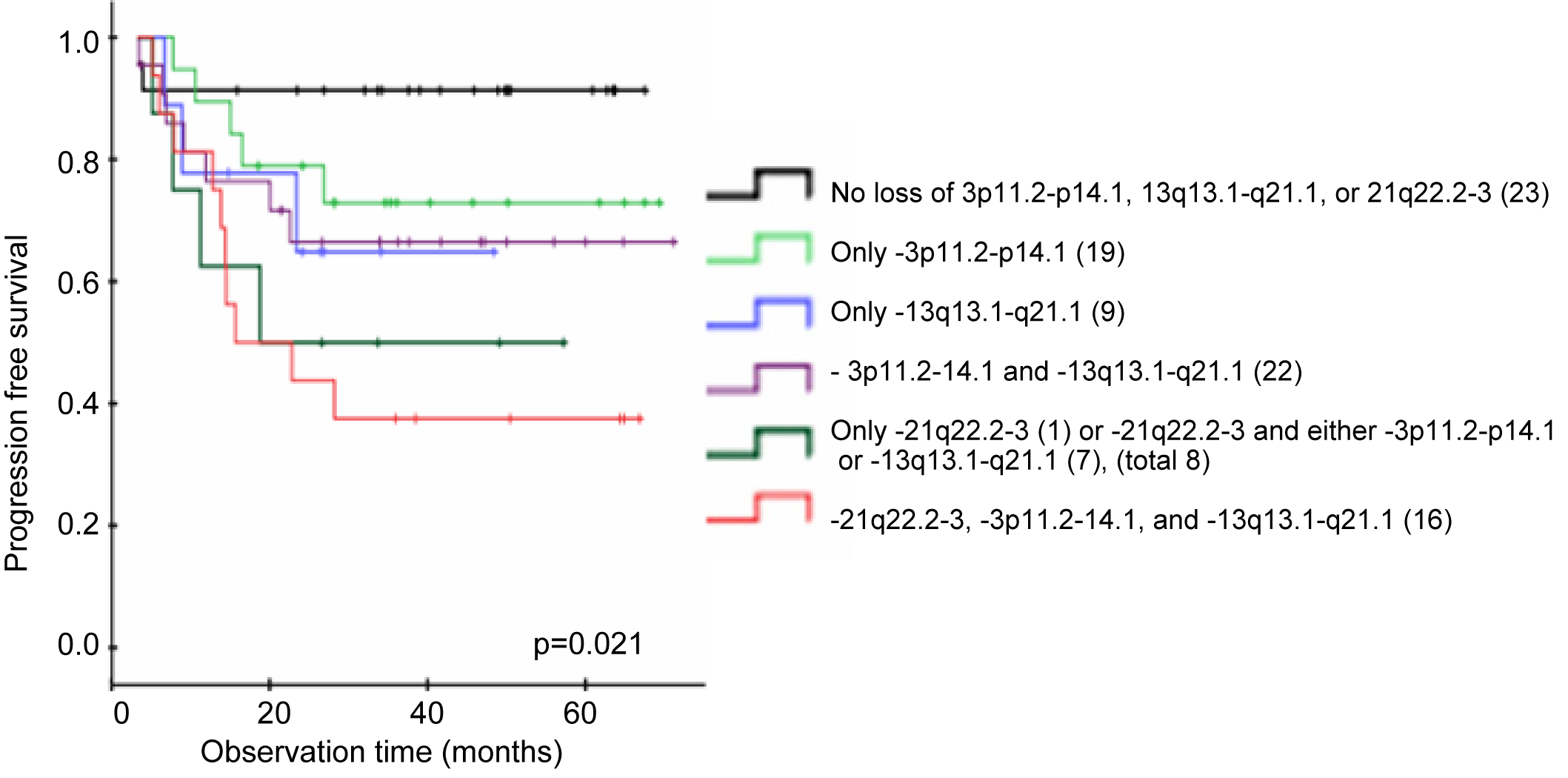

Supplement: Figure S3 — Clinical outcome for patients with different combinations of predictive losses. Kaplan-Meier curves showing progression free survival after chemoradiotherapy of 97 cervical cancer patients with different combinations of 3p11.2-p14.1, 13q13.1-q21.1, and 21q22.2-3 loss. The different combinations and number of patients in each group are listed (right). P-value in log-rank test is indicated. (0.24 MB TIF) [file pgen.1000719.s003.tif]

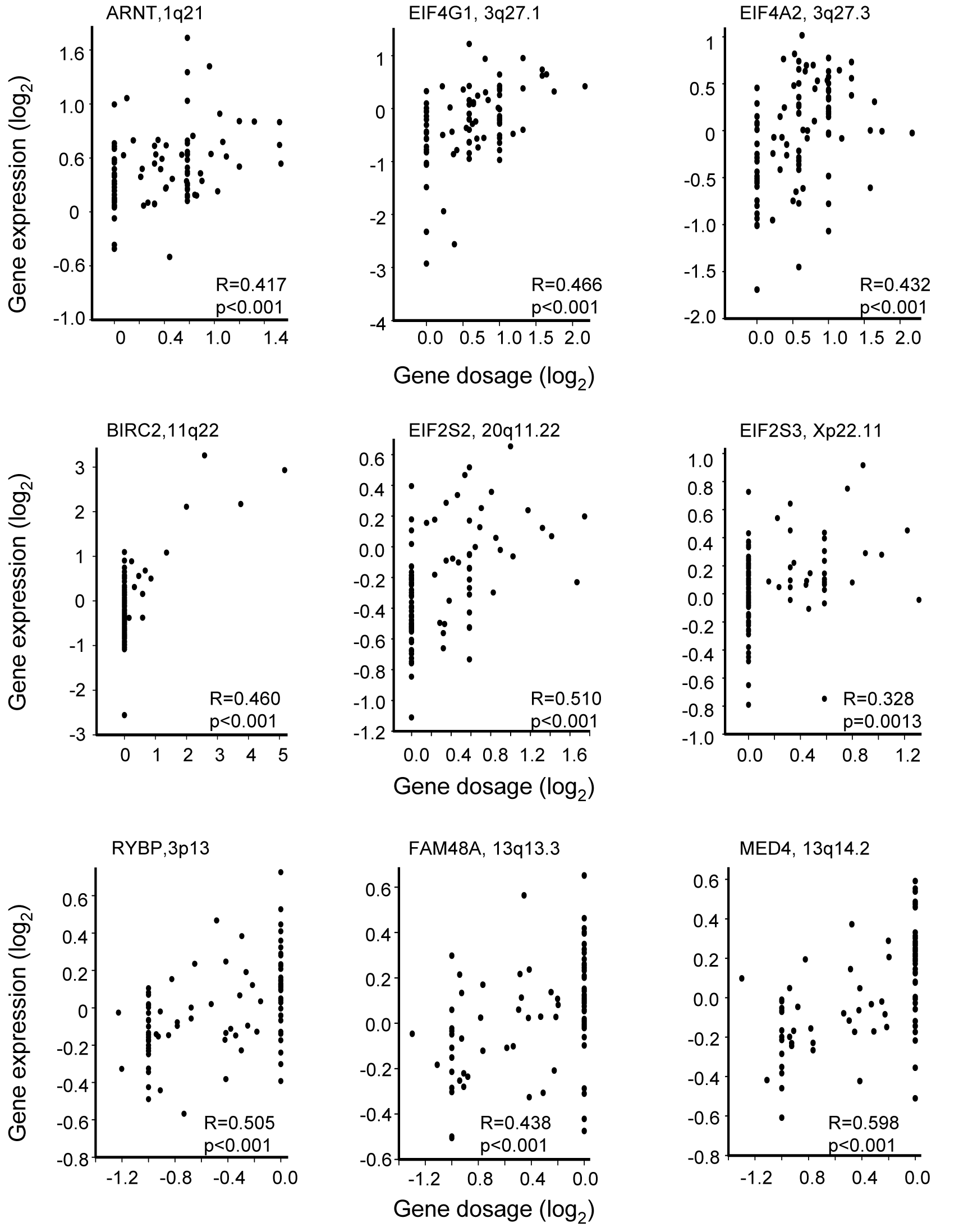

Supplement: Figure S4 — Correlations between gene dosage and expression. Typical correlation plots of gene dosage and expression for 9 correlating genes within the recurrent and predictive regions; 6 with gain and 3 with loss. Spearman's rank correlation analysis on semi-discrete data was performed, for which amplitudes lower than 1.1 were set to 1 for gains and amplitudes higher than 0.9 were set to 1 for losses. Correlation coefficient (R) and p-value are indicated. (0.27 MB TIF) [file pgen.1000719.s004.tif]
