# Supplementary material for: Gene Dosage, Expression, and Ontology Analysis Identifies Driver Genes in the Carcinogenesis and Chemoradioresistance of Cervical Cancer
Source: PLoS Genet. 2009 Nov 13;5(11):e1000719. doi: 10.1371/journal.pgen.1000719 (PMC2768783; doi:10.1371/journal.pgen.1000719)
Supplement: Table S1 — Recurrent high-level amplifications and homozygous deletions in locally advanced cervical cancer. (0.03 MB PDF) [file pgen.1000719.s005.pdf]

Table S1. Recurrent high level amplifications and homozygous deletions in locally advanced cervical cancer.

Peak region[a] (Cytoband)	Peak region[a] (MB)	Freq.[b] (%)	Max./min. gene dosage[c] (copy no.)	Correlating genes[d]
**Recurrent high level amplification**				
3q26.1-qter	166.2-199.5	8	4.5 (9)	*PDCD10, PHC3, ZNF639, FXR1, PARL, DVL3, ABCF3, ALG3, EIF4G1, SFRS10, DGKG, EIF4A2, RFC4, CCDC50, PPP1R2, PAK2, NCBP2, DLG1, BDH1, FLYTTD1*
5p15.2-pter	1.0-12.1	8	4 (15)	*CLPTM1L , MED10, FASTKD3, CCT5, DAP*
9p24.1-2	2.7-6.0	4	13.5 (27)	*KIAA0020, RCL1*
11q13.2-3	68.6-70.6	4	10 (20)	*FADD*
11q22.1-2	100.2-102.0	5	36 (72)	*YAP1, BIRC3, BIRC2*
20q11.21-22	30.0-33.0	5	3.4 (9)	*POFUT1, KIF3B, MAPRE1, SNTA1, EIF2S2, AHCY*
21q22.11-2	32.9-39.6	4	7.5 (15)	*TTC3, BRWD1*
**Homozygous deletion[e]**				
5q13.2	67.4-71.7	1	0 (0)	*SMN2*
6p21.1-p12.1	44.1-54.1	1	0 (0)	-
8q24.23	136.6-139.3	1	0 (0)	-
9p21.1-3	22.6-29.6	1	0 (0)	*MOBKL2B*
10q23.31	88.2-92.1	3	0 (0)	-
13q34	111.7-114.1	1	0 (0)	-

[a]Peak region of high level amplifications is the region with more than 25% higher amplitude than surrounding region. Peak region of homozygote deletions is the region with a gene dosage of zero.
[b]Frequency is the median percentage of tumors with the alteration.
[c]Gene dosage is absolute DNA copy number divided by ploidy. Maximum (gain) or minimum (loss) gene dosage and the corresponding copy number are listed.
[d]Genes within the peak region showing a correlation between gene dosage and expression are ordered by DNA location.
[e]Homozygote deletions were seen in only few tumors and were not detected as recurrent in statistical analysis.
