# Supplementary material for: Gene Dosage, Expression, and Ontology Analysis Identifies Driver Genes in the Carcinogenesis and Chemoradioresistance of Cervical Cancer
Source: PLoS Genet. 2009 Nov 13;5(11):e1000719. doi: 10.1371/journal.pgen.1000719 (PMC2768783; doi:10.1371/journal.pgen.1000719)
Supplement: Table S2 — Relationships among Illumina, cDNA, and gene dosage data for correlating genes. (0.07 MB PDF) [file pgen.1000719.s006.pdf]

**Table S2.** Relationships between Illumina, cDNA, and gene dosage data for correlating genes[a].

| Reporter ID | IlluminaID | Gene | cDNA vs gene dosage | | cDNA vs gene dosage | | cDNA vs Illumina | | Illumina vs gene dosage | |
|---|---|---|---|---|---|---|---|---|---|---|
| | | | 95 patients | | 52 patients | | | | | |
| | | | R | p | R | p | R | p | R | p |
| 129563 | ILMN_1762582 | *ARNT* | 0.407 | 0.000 | 0.403 | 0.004 | 0.412 | 0.003 | 0.411 | 0.003 |
| 814158 | ILMN_1669113 | *ATF5* | 0.347 | 0.001 | 0.520 | 0.000 | 0.812 | 0.000 | 0.488 | 0.000 |
| 825312 | ILMN_1772929 | *ATP5J* | 0.369 | 0.002 | 0.421 | 0.007 | 0.611 | 0.000 | 0.368 | 0.010 |
| 877832 | ILMN_1853837 | *BCAP31* | 0.327 | 0.001 | 0.225 | 0.111 | 0.866 | 0.000 | 0.280 | 0.047 |
| 782748 | ILMN_1708485 | *BIN3* | 0.502 | 0.000 | 0.577 | 0.000 | 0.345 | 0.013 | 0.415 | 0.003 |
| 34852 | ILMN_1768194 | *BIRC2* | 0.475 | 0.000 | 0.467 | 0.000 | 0.697 | 0.000 | 0.573 | 0.000 |
| 201890 | ILMN_2405684 | *BIRC3* | 0.476 | 0.000 | 0.445 | 0.001 | 0.636 | 0.000 | 0.237 | 0.091 |
| 249618 | ILMN_1752802 | *CLPTM1L* | 0.367 | 0.001 | 0.484 | 0.002 | 0.517 | 0.000 | 0.701 | 0.000 |
| 343352 | ILMN_1779530 | *COG6* | 0.403 | 0.000 | 0.510 | 0.000 | 0.622 | 0.000 | 0.451 | 0.002 |
| 897971 | ILMN_1699112 | *COPB1* | 0.345 | 0.001 | 0.579 | 0.000 | 0.468 | 0.000 | 0.556 | 0.000 |
| 884480 | ILMN_1798189 | *COX7C* | 0.500 | 0.000 | 0.341 | 0.017 | 0.609 | 0.000 | 0.407 | 0.004 |
| 814381 | ILMN_2112493 | *DAP* | 0.404 | 0.000 | 0.565 | 0.000 | 0.790 | 0.000 | 0.621 | 0.000 |
| 487082 | ILMN_1706498 | *DSE* | 0.378 | 0.000 | 0.365 | 0.010 | 0.586 | 0.000 | 0.444 | 0.002 |
| 22918 | ILMN_1768127 | *EBNA1BP2* | 0.293 | 0.004 | 0.242 | 0.086 | 0.696 | 0.000 | 0.352 | 0.012 |
| 469151 | ILMN_1798014 | *EIF2S2* | 0.508 | 0.000 | 0.571 | 0.000 | 0.444 | 0.001 | 0.439 | 0.001 |
| 810237 | ILMN_1665717 | *EIF2S3* | 0.427 | 0.000 | 0.248 | 0.076 | 0.492 | 0.000 | 0.320 | 0.021 |
| 307532 | ILMN_1685722 | *EIF4A2* | 0.434 | 0.000 | 0.459 | 0.000 | 0.633 | 0.000 | 0.552 | 0.000 |
| 25988 | ILMN_2370772 | *EIF4G1* | 0.468 | 0.000 | 0.314 | 0.038 | 0.636 | 0.000 | 0.446 | 0.002 |
| 809453 | ILMN_1802376 | *FAM48A* | 0.415 | 0.000 | 0.349 | 0.016 | 0.492 | 0.000 | 0.344 | 0.018 |
| 133158 | ILMN_1750160 | *FASTKD3* | 0.619 | 0.000 | 0.782 | 0.000 | 0.645 | 0.000 | 0.608 | 0.000 |
| 82171 | ILMN_1687940 | *FOXO3* | 0.335 | 0.002 | 0.315 | 0.033 | 0.618 | 0.000 | 0.359 | 0.015 |
| 289551 | ILMN_2389273 | *FXR1* | 0.435 | 0.000 | 0.530 | 0.000 | 0.752 | 0.000 | 0.554 | 0.000 |
| 127509 | ILMN_1789702 | *GBE1* | 0.347 | 0.001 | 0.271 | 0.057 | 0.465 | 0.000 | 0.365 | 0.009 |
| 754085 | ILMN_1745798 | *GTF2F2* | 0.344 | 0.001 | 0.473 | 0.000 | 0.700 | 0.000 | 0.480 | 0.000 |
| 811942 | ILMN_2157957 | *GTF2H1* | 0.341 | 0.001 | 0.323 | 0.021 | 0.449 | 0.000 | 0.215 | 0.130 |
| 256664 | ILMN_2200331 | *H2AFX* | 0.388 | 0.000 | 0.305 | 0.036 | 0.495 | 0.000 | 0.332 | 0.021 |
| 502669 | ILMN_1767747 | *HDAC2* | 0.439 | 0.000 | 0.471 | 0.000 | 0.521 | 0.000 | 0.652 | 0.000 |
| 1606829 | ILMN_1764396 | *HDAC4* | 0.357 | 0.001 | 0.397 | 0.005 | 0.395 | 0.004 | 0.301 | 0.036 |
| 843319 | ILMN_1792497 | *HRB* | 0.425 | 0.000 | 0.466 | 0.000 | 0.292 | 0.036 | 0.415 | 0.002 |
| 810942 | ILMN_1802706 | *IDH3G* | 0.428 | 0.000 | 0.486 | 0.000 | 0.789 | 0.000 | 0.275 | 0.051 |
| 795282 | ILMN_1664641 | *MED4* | 0.568 | 0.000 | 0.502 | 0.000 | 0.445 | 0.001 | 0.446 | 0.003 |
| 131653 | ILMN_2371964 | *MRPS12* | 0.455 | 0.000 | 0.521 | 0.000 | 0.785 | 0.000 | 0.548 | 0.000 |
| 810979 | ILMN_1815043 | *MRPS2* | 0.365 | 0.001 | 0.333 | 0.018 | 0.495 | 0.000 | 0.507 | 0.000 |
| 470216 | ILMN_1727080 | *MYO6* | 0.373 | 0.000 | 0.219 | 0.122 | 0.298 | 0.032 | 0.373 | 0.007 |
| 26711 | ILMN_1720442 | *NCBP2* | 0.494 | 0.000 | 0.442 | 0.002 | 0.590 | 0.000 | 0.682 | 0.000 |
| 753457 | ILMN_1728810 | *NDUFS1* | 0.431 | 0.000 | 0.405 | 0.004 | 0.383 | 0.005 | 0.476 | 0.000 |
| 795439 | ILMN_2323491 | *NUP62* | 0.318 | 0.002 | 0.438 | 0.002 | 0.281 | 0.044 | 0.451 | 0.001 |
| 134439 | ILMN_1712687 | *PAK2* | 0.304 | 0.003 | 0.245 | 0.093 | 0.414 | 0.002 | 0.568 | 0.000 |
| 137836 | ILMN_2269002 | *PDCD10* | 0.588 | 0.000 | 0.646 | 0.000 | 0.775 | 0.000 | 0.705 | 0.000 |
| 80374 | ILMN_1772369 | *PDHA1* | 0.327 | 0.001 | 0.177 | 0.214 | 0.743 | 0.000 | 0.207 | 0.144 |
| 248454 | ILMN_1815261 | *PDIA4* | 0.357 | 0.001 | 0.289 | 0.049 | 0.621 | 0.000 | 0.445 | 0.002 |
| 454475 | ILMN_1814074 | *PHKA2* | 0.470 | 0.000 | 0.298 | 0.036 | 0.656 | 0.000 | 0.418 | 0.003 |
| 112131 | ILMN_1776076 | *POFUT1* | 0.303 | 0.003 | 0.476 | 0.000 | 0.662 | 0.000 | 0.457 | 0.000 |
| 2191807 | ILMN_1773613 | *POU2F3* | 0.399 | 0.000 | 0.257 | 0.113 | 0.295 | 0.052 | 0.120 | 0.431 |
| 769657 | ILMN_1683044 | *PPP1R2* | 0.524 | 0.000 | 0.551 | 0.000 | 0.558 | 0.000 | 0.637 | 0.000 |
| 125148 | ILMN_1813766 | *RCL1* | 0.603 | 0.000 | 0.492 | 0.000 | 0.719 | 0.000 | 0.515 | 0.000 |
| 853151 | ILMN_1651850 | *RPS16* | 0.440 | 0.000 | 0.418 | 0.002 | 0.042 | 0.766 | 0.169 | 0.236 |
| 795453 | ILMN_1721842 | *RYBP* | 0.494 | 0.000 | 0.606 | 0.000 | 0.522 | 0.000 | 0.480 | 0.000 |
| 450131 | ILMN_1752111 | *SMARCAL1* | 0.437 | 0.000 | 0.614 | 0.000 | 0.521 | 0.000 | 0.625 | 0.000 |
| 295255 | ILMN_1788211 | *SNX19* | 0.422 | 0.000 | 0.417 | 0.006 | 0.671 | 0.000 | 0.440 | 0.003 |
| 884657 | ILMN_1738938 | *TIMM8B* | 0.432 | 0.000 | 0.649 | 0.000 | 0.717 | 0.000 | 0.587 | 0.000 |
| 878744 | ILMN_1747146 | *TSG101* | 0.538 | 0.000 | 0.554 | 0.000 | 0.632 | 0.000 | 0.549 | 0.000 |
| 739126 | ILMN_1697777 | *TSTA3* | 0.387 | 0.000 | 0.336 | 0.020 | 0.336 | 0.000 | 0.222 | 0.128 |
| 346292 | ILMN_1675674 | *UBE4B* | 0.304 | 0.003 | 0.460 | 0.001 | 0.661 | 0.000 | 0.367 | 0.011 |
| 206545 | ILMN_2174884 | *XPO7* | 0.400 | 0.000 | 0.434 | 0.001 | 0.742 | 0.000 | 0.614 | 0.000 |
| 126702 | ILMN_1792990 | *ZNF202* | 0.333 | 0.001 | 0.355 | 0.013 | 0.289 | 0.038 | 0.387 | 0.006 |

[a]Data for the genes in Figure 5 are shown, except for SLC25A6 which was not included in the Illumina data set.
